# Safety of PD-1/PD-L1 Inhibitors Combined With Palliative Radiotherapy and Anti-Angiogenic Therapy in Advanced Hepatocellular Carcinoma

**DOI:** 10.3389/fonc.2021.686621

**Published:** 2021-05-19

**Authors:** Liting Zhong, Dehua Wu, Weiwei Peng, Hailong Sheng, Yazhi Xiao, Xuebing Zhang, Yuli Wang

**Affiliations:** ^1^ Department of Radiation Oncology, Nanfang Hospital, Southern Medical University, Guangzhou, China; ^2^ Department of Oncology, Ganzhou People’s Hospital (The Affiliated Ganzhou Hospital of Nanchang University), Ganzhou, China

**Keywords:** hepatocellular carcinoma, immunotherapy, immune checkpoint inhibitor, radiotherapy, anti-angiogenic therapy, targeted angiogenesis therapy

## Abstract

**Background:**

Previous studies have explored cancer immunotherapy with radiotherapy or anti-angiogenic therapy, but no trials have reported a triple therapy approach. This study aimed to investigate safety and clinical outcome of PD-1**/**PD-L1 inhibitors combined with palliative radiotherapy and targeted angiogenesis therapy in hepatocellular carcinoma (HCC) of Barcelona Clinic Liver Cancer (BCLC) stage C.

**Methods:**

Consecutive patients (n=16) treated with PD-1**/**PD-L1 inhibitors combined with radiotherapy and anti-angiogenic therapy in a bi-institutional cohort between July 2017 and December 2020 were retrospectively included. Radiotherapy was conducted within 14 days of the first administration of immunotherapy. The primary endpoint was treatment-related adverse event (TRAE).

**Results:**

The median follow-up was 383 days. Fifteen patients (93.8%) experienced at least 1 TRAE. The most common TRAEs of any grade were rash (25%), diarrhea (25%), aspartate aminotransferase increase (18.8%), alanine transaminase increase (18.8%), decreased appetite (18.8%), and fatigue (18.8%). Grade 3/4 TRAEs occurred in 4 patients (25%) and finally led to treatment interruption. No patient death was attributed to treatment. No specific events were responsible for the addition of radiotherapy. Six patients showed partial response, 7 showed stable disease, and 2 showed progressive disease. The objective response rate and disease control rate were 40.0% (95% CI 16.3%–67.7%) and 86.7% (95% CI 59.5%–98.3%), respectively. Moreover, the median progression-free survival was 140 days. Patients had a median overall survival of 637 days, and the estimated rates of survival at 6 and 12 months were 92.3% and 75.5%, respectively.

**Conclusion:**

PD-1**/**PD-L1 inhibitors combined with palliative radiotherapy and anti-angiogenic therapy appear to be safe, with no unexpected adverse events. Additional studies exploring the clinical benefit are warranted.

## Introduction

Hepatocellular carcinoma (HCC) is a type of cancer that is common worldwide and the fourth most common cause of cancer-related deaths worldwide (1). Most HCC patients are diagnosed at Barcelona Clinic Liver Cancer (BCLC) stage C, are unresponsive to curative therapies, and exhibit a very poor prognosis. Cancer immunotherapy, especially programmed death receptor-1**/**programmed death ligand-1 (PD-1**/**PD-L1) inhibitor, has demonstrated promising treatment efficacy against HCC in Phase 1/2 studies (2, 3). However, subsequent Phase 3 trials testing nivolumab versus sorafenib in the first line and pembrolizumab versus placebo in the second line both failed to meet their primary survival endpoints (4, 5)

It is well known that only a minority of patients currently benefit from immune checkpoint inhibitor (ICI) monotherapy. Intrinsic and acquired resistance to ICIs has focused research on new combination therapy approaches. Combination therapies involving PD-1**/**PD-L1 inhibitors are being studied for a variety of malignancies, such as non–small cell lung cancer (NSCLC), renal cell carcinoma, and endometrial cancer (6, 7). For unresectable HCC, a combination of atezolizumab plus bevacizumab succeeded in a front-line Phase 3 trial (IMbrave150). Compared with sorafenib, atezolizumab plus bevacizumab improved both co-primary endpoints overall survival (median OS, not evaluable *vs* 13.2 months; HR, 0.58) and progression-free survival (median PFS, 6.8 vs 4.3; HR, 0.59), showed a good safety profile and improved quality of life (8). Thus, atezolizumab plus bevacizumab became the new standard of first-line therapy. Furthermore, lenvatinib plus pembrolizumab and camrelizumab in combination with apatinib in HCC have also received encouraging results from a Phase 1b and 2 study, respectively (9, 10).

In addition to combinations of PD-1**/**PD-L1 blockade with targeting angiogenesis therapy, mounting evidence supports the role of radiotherapy in potentiating tumor immunity. Radiation can elicit an immune-stimulatory form of cell death, termed immunogenic cell death (ICD), leading to the release of cytokines and damage-associated molecular patterns (DAMPs). These signals favor the recruitment of antigen-presenting cells (APCs) and enhance their phagocytic activity, processing of tumor-associated antigens (TAAs), and cross-presentation of antigenic peptides on major histocompatibility complex class I (MHC I). Cross-presentation of tumor antigens can lead to subsequent priming and trafficking of tumor-specific T lymphocytes into the tumor microenvironment (11). The initial results of combining radiotherapy with immunomodulatory agents have generated promising data in pre-clinical studies (12, 13), which has, in turn, led to a large number of radiotherapy and immunotherapy clinical trials (14, 15). The PACIFIC trial showed significantly longer overall survival when durvalumab was given after standard chemoradiotherapy in patients with unresected stage III NSCLC (16). Chiang et al. reported a 100% objective response rate (ORR) in 5 patients receiving stereotactic body radiotherapy (SBRT) followed by nivolumab for large unresectable HCC, with acceptable toxicity (17). A prospective trial showed that a combination of Y90-radioembolization with nivolumab had an optimistic ORR of 31% in advanced HCC, with only 11% of patients experienced grade 3/4 treatment-related adverse events (18). Several ongoing clinical trials are being conducted to investigate the combined approach of ICI and external beam radiotherapy for patients with HCC.

Thus far, combinations of any two of the following treatment approaches, namely, radio-, immune-, or anti-angiogenic therapies, have shown therapeutic potential in improving treatment outcomes (19). Therefore, a trimodal approach combining cancer immunotherapy with anti-angiogenic therapy and radiotherapy offers an innovative and interesting therapeutic strategy for the treatment of cancer (20, 21). However, there has been no detailed investigation of PD-1**/**PD-L1 inhibitors in combination with radiotherapy and targeting angiogenesis therapy for HCC of BCLC stage C. Herein, we report a retrospective study of the combined approach with an aim to provide the data of the safety and efficacy.

## Materials and Methods

### Patients

In this retrospective study, we identified HCC patients who received PD-1**/**PD-L1 inhibitors plus radiotherapy and anti-angiogenic therapy at Nanfang Hospital Southern Medical University and Ganzhou People’s Hospital (The Affiliated Ganzhou hospital of Nanchang University) between July 2017 and December 2020. Radiation was done within 14 days of the first administration of immunotherapy. We included any radiation concept with regard to the fractionation scheme, radiation dose, and radiation site. Possible fractionation schemes were the conventional fractionated radiation therapy, which was between 1.8 and 2 Gy single dose with 5 fractions per week; the hypofractionated radiotherapy with higher irradiation doses between 2.5 and 4 Gy single dose and less fractions; and SBRT. Targeted agents containing vascular endothelial growth factor (VEGF) blockade (bevacizumab and apatinib) and other anti-angiogenic drugs such as multi-tyrosine kinase inhibitors (lenvatinib, sorafenib, and regorafenib) were initiated concurrently with PD-1**/**PD-L1 inhibitors. Patients were excluded if they had received liver transplantation or have other malignant tumors. Patients with hepatitis B virus (HBV) infection were covered with anti-viral therapy before treatment. Ethical approval for the study was granted by the Institutional Ethics Committee of two hospitals, and the study complied with the Declaration of Helsinki. The need for obtaining patient consent was waived due to the retrospective nature of our study.

### Data Collection

We extracted data on sex, age, HBV infection status, Eastern Cooperative Oncology Group (ECOG) score, alpha-fetoprotein (AFP) level, PD-1**/**PD-L1 inhibitor substance, targeted agents, radiotherapy, and prior therapy from medical records. The primary endpoint was treatment-related adverse event (TRAE). TRAE were extracted in oncology clinic visit notes or medical records and reviewed according to the US National Cancer Institute (NCI) Common Terminology Criteria for Adverse Events (CTCAE v4.03). In the event of multiple instances of the same toxicity, the maximum grade per patient for the given category was taken.

Treatment response was evaluated by contrast-enhanced computed tomography or magnetic resonance imaging every six to eight weeks after the first cycle of PD-1**/**PD-L1 inhibitors. Tumor efficacy was assessed as either complete (CR) or partial (PR) response, as well as stable (SD) or progressive (PD) disease, according to the Response Evaluation Criteria in Solid Tumors (RECIST) v.1.1. The objective response rate (ORR) was defined by the presence of either a CR or PR. The disease control rate (DCR) was defined as the sum of CR, PR, and SD. Duration of response (DOR) was calculated from the date of first response to progression or death. Overall survival (OS) was defined from the first cycle of PD-1**/**PD-L1 inhibitor administration to the date of death or last contact. Progression-free survival (PFS) was defined as the time from the first treatment administration to the date of PD, death, or last follow-up.

### Statistical Analyses

All statistical analyses were performed using SPSS Statistics for Windows, Version 26.0 (SPSS Inc., Chicago, IL, USA). Graphs were plotted by GraphPad Prism 8.0.2. ORR and DCR were calculated with 95% CI using the Clopper-Pearson method. DOR, OS, and PFS were estimated using the Kaplan-Meier method. Log-rank tests were used to analyze survival. A double-tailed *P-*value <0.05 was considered significant.

## Results

### Patient Characteristics and Treatments

Overall, 16 patients with HCC of BCLC stage C (12 at Nanfang Hospital and 4 at Ganzhou People’s Hospital) were enrolled in the study. Details on patient and treatment characteristics are summarized in **Tables 1**–**3**. The median patient age was 51.5 years (range, 21–74 years), and most patients were male (87.5%). At baseline, ECOG scores of 0, 1, and 2 were reported for 6.3%, 62.5%, and 31.3% of patients, respectively. 56.3% of patients had an AFP level ≧ 400 ng/mL. Child-Pugh class A was noted in 81.3% of patients and class B in 18.8%. There were 13 patients (81.3%) with HBV. Proportions of patients with portal vein invasion and extrahepatic spread were 50.0% and 75.0%, respectively.

**Table 1:** Patient characteristics and treatments.

Characteristics	All (%) (n=16)
Age (year)	
Median(range)	51.5(21-74)
Gender	
Male	14 (87.5)
Female	2 (12.5)
ECOG score	
0	1 (6.3)
1	10 (62.5)
2	5 (31.3)
Serum AFP level, ng/mL	
<400	7 (43.8)
≧400	9 (56.3)
Child-Pugh class	
A	13 (81.3)
B	3 (18.8)
HBV infection	13 (81.3)
Portal vein invasion	8 (50.0)
Extrahepatic spread	11 (68.8)
Duration of PD-1**/**PDL-1 inhibitors^a^ (day)	
Median(range)	153 (0-488)
Course of PD-1**/**PDL-1 inhibitors	
Median(range)	6.5(1-14)
Radiotherapy site	
Liver	8 (50.0)
Bone	5 (31.3)
Brain	1 (6.3)
Inferior vena cava tumor thrombus	1 (6.3)
Soft tissue of the lumbar spine	1 (6.3)
Radiotherapy dose (Gray)	
Median(range)	43.5(30-60)
Radiotherapy technique	
SBRT	6 (37.5)
Hypofractionated radiotherapy	7 (43.8)
Conventional radiotherapy	3 (18.8)
Prior treatment	
No	4 (25.0)
Hepatectomy	6 (37.5)
Radiofrequency ablation	5 (31.3)
TACE	9 (56.3)
Targeted agents^b^	7 (43.8)

a Including camrelizumab (n=5), sintilimab (n=3), tislelizumab (n=2), pembrolizumab (n=1), nivolumab (n=1), toripalimab (n=1), atezolizumab (n=1), sequential therapy (n=2).

b Including lenvatinib (n=2), sorafenib (n=1), apatinib (n=1), sequential therapy (n=3).

ECOG, Eastern Cooperative Oncology Group; AFP, alpha-fetoprotein; HBV, hepatitis B virus; SBRT, stereotactic body radiotherapy; TACE, trans-arterial chemo-embolization.

**Table 2:** Detailed characteristics of patients.

	Age	Gender	ECOG	AFP ng/mL	Child-Pugh class	HBV	Portal vein invasion	Extrahepatic spread	Metastatic site
1	51	Male	1	429.5	A	No	No	Yes	Lung, lymph nodes
2	72	Male	1	2.4	A	Yes	Yes	No	–
3	71	Male	1	1.7	A	Yes	Yes	Yes	Lung
4	62	Male	1	507.2	B	Yes	No	Yes	Lymph nodes
5	54	Male	2	514.9	A	Yes	Yes	No	–
6	60	Male	0	38.7	A	Yes	Yes	No	–
7	42	Male	1	56.1	A	No	No	Yes	Bone
8	52	Male	1	323.7	A	Yes	Yes	No	–
9	74	Male	2	5.0	A	No	No	Yes	Bone, lymph nodes
10	51	Male	1	44880.5	A	Yes	Yes	Yes	Lung, bone
11	43	Male	2	1664.3	B	Yes	No	Yes	Lung, bone
12	47	Male	2	12687.0	A	Yes	No	Yes	Lung, bone, lymph nodes, adrenal
13	46	Female	2	6223.0	A	Yes	Yes	Yes	Lung, bone
14	21	Male	1	3399.0	A	Yes	No	Yes	Lung, brain
15	51	Male	1	5372.0	B	Yes	No	Yes	Bone, lymph nodes
16	65	Female	1	355.1	A	Yes	Yes	Yes	Adrenal,inferior vena cava tumor thrombus

ECOG, Eastern Cooperative Oncology Group; AFP, alpha-fetoprotein; HBV, hepatitis B virus.

**Table 3:** Treatment details, toxicities, and outcome of patients.

	PD-1/PDL-1 inhibitor(course)	Targeted agent	RT site	RT dose(Gy)	RT technique	Prior treatment	Toxicity	Tumor response	Status
1	Sintilimab(4)	Sorafenib	Liver	40	SBRT	No	Grade 2 decreased appetite	PD	Alive
2	Tislelizumab(7)	Sorafenib, regorafenib	Liver	60	Hypofractionated RT	Hepatectomy, radiofrequency ablation	Grade 1 rash, Grade 2 hypertension	SD	Alive
3	Sintilimab, camrelizumab(14)	Regorafenib, apatinib	Liver	54	Hypofractionated RT	Radiofrequency ablation,TACE, sorafenib	Grade 2 rash	PR	Alive
4	Camrelizumab(6)	Lenvatinib	Liver	45	Conventional RT	TACE, lenvatinib	Grade 2 diarrhea, decreased appetite, fatigue	PR	Alive
5	Sintilimab(10)	Sorafenib, regorafenib	Liver	45	Conventional RT	TACE, sorafenib	Grade 2 nausea	SD	Alive
6	Camrelizumab(3)	Apatinib, regorafenib	Liver	54	Hypofractionated RT	Radiofrequency ablation,TACE	Grade 3 rash; Grade 2 infusion-related reaction; Grade 2 pruritus; Grade 2 blood bilirubin/aspartate aminotransferase/alanine transaminase increase	PR	Alive
7	Pembrolizumab(6)	Apatinib	Liver	50	SBRT	Apatinib	Grade 1 diarrhea	PD	Death
8	Nivolumab(7)	Sorafenib	Liver	45	SBRT	No	Grade 2 alanine transaminase increase; Grade 3 aspartate aminotransferase increase	PR	Alive
9	Atezolizumab(1)	Bevacizumab	Bone	36	Hypofractionated RT	No	–	–	Alive
10	Tislelizumab(3)	Lenvatinib	Soft tissue of the lumbar spine	36	Hypofractionated RT	TACE	Grade 2 dental ulcer,diarrhea,decreased appetite,weight decrease	PR	Alive
11	Toripalimab(4)	Lenvatinib, regorafenib	Bone	40	SBRT	No	Grade 1 fatigue; Grade 2 aspartate aminotransferase increase; Grade 3 alanine transaminase increase	SD	Death
12	Camrelizumab(9)	Sorafenib, lenvatinib	Bone	40	Conventional RT	Hepatectomy,TACE, sorafenib	Grade 1 fatigue	SD	Death
13	Camrelizumab(4)	Lenvatinib	Bone	35	SBRT	Hepatectomy, radiofrequency ablation,TACE, sorafenib	Grade 2 rash, pruritus, nausea	SD	Death
14	Tislelizumab, atezolizumab(10)	Lenvatinib, regorafenib	Brain	42	SBRT	Hepatectomy	Grade 2 diarrhea	SD	Alive
15	Camrelizumab(10)	Sorafenib, regorafenib	Bone	30	Hypofractionated RT	Hepatectomy,TACE	Grade 2 dental ulcer	SD	Alive
16	Sintilimab(8)	Regorafenib	Inferior vena cava tumor thrombus	55	Hypofractionated RT	Hepatectomy, radiofrequency ablation,TACE, sorafenib	Grade 4 gastrointestinal hemorrhage	PR	Alive

RT, radiotherapy; SBRT, stereotactic body radiotherapy; TACE, trans-arterial chemo-embolization; PR, partial response; SD, stable disease; PD, progressive disease.

The median duration of treatment from the initiation of immunotherapy was 153 days (range 0–488 days) with a median of 6.5 courses (range 1–14). Most patients received camrelizumab (n=5). Other PD-1**/**PD-L1 inhibitors include sintilimab (n=3), tislelizumab (n=2), pembrolizumab (n=1), nivolumab (n=1), toripalimab (n=1), and atezolizumab (n=1). Two patients were treated with sequential immunotherapy. All patients received a concomitant combination of anti-angiogenic agents, including lenvatinib (n=4), sorafenib (n=1), regorafenib (n=1), apatinib (n=1), bevacizumab (n=1), and sequential therapy (n=8). The majority received radiotherapy at the liver (n=8), followed by the bone (n=5), brain (n=1), inferior vena cava tumor thrombus (n=1), and soft tissue of the lumbar spine (n=1). The median radiation dose delivered was 43.5 Gy (range 30–60 Gy). The type of radiotherapy received were: stereotactic body radiotherapy (SBRT) (n=6), hypofractionated radiotherapy (n=7), and conventional radiotherapy (n=3).

### Safety

Overall, 15 patients (93.8%) experienced at least 1 TRAE (**Table 4**). The most common TRAEs of any grade were rash (25%), diarrhea (25%), aspartate aminotransferase increase (18.8%), alanine transaminase increase (18.8%), decreased appetite (18.8%), and fatigue (18.8%). Grade 3/4 TRAEs occurred in 4 patients (25%) and finally led to treatment interruption; these included rash, aspartate aminotransferase increase, alanine transaminase increase, and gastrointestinal hemorrhage. Overall, there were no radiation dose reductions. TRAE led to treatment discontinuation of immunotherapy (camrelizumab) in 1 patient (6.3%) due to an infusion-related reaction. No patient death was attributed to treatment. No specific events were responsible for the addition of radiotherapy.

**Table 4:** Treatment Related Adverse Events.

Adverse Events	Any Grade, n (%)	Grade 3/4, n (%)
Rash	4(25.0%)	1(6.3%)
Diarrhea	4(25.0%)	0
Aspartate aminotransferase increase	3(18.8%)	1(6.3%)
Alanine transaminase increase	3(18.8%)	1(6.3%)
Decreased appetite	3(18.8%)	0
Fatigue	3(18.8%)	0
Pruritus	2(12.5%)	0
Dental ulcer	2(12.5%)	0
Nausea	2(12.5%)	0
Hypertension	1(6.3%)	0
Infusion-related reaction	1(6.3%)	0
Gastrointestinal hemorrhage	1(6.3%)	1(6.3%)
Blood bilirubin increase	1(6.3%)	0
Weight decrease	1(6.3%)	0

TRAEs in the liver-directed radiotherapy and non liver-directed radiotherapy groups are shown in **Table S1**. In the 8 patients who received liver-directed radiotherapy, 2 (25%) patients experienced abnormal hepatic function (1 with aspartate aminotransferase and alanine transaminase increase, 1 with aspartate aminotransferase, alanine transaminase, and blood bilirubin increase). There was 1 (12.5%) patient with aspartate aminotransferase and alanine transaminase increase in the non liver-directed radiotherapy group. Grade 3/4 TRAEs were observed in 2 (25%) patients in the liver-directed radiotherapy group (rash and aspartate aminotransferase increase) and in 2 (25%) patients in the non liver-directed radiotherapy group (alanine transaminase increase and gastrointestinal hemorrhage), leading to treatment interruption.

### Clinical Outcome

As shown in **Table 5**, 15 patients were evaluated for treatment efficacy. Treatment response was not assessed in 1 patient who received the first course of immunotherapy. The median time to evaluate from the initiation of immunotherapy was 55 days (range 35–108 days). According to the independent assessment with RECIST 1.1, there were 6 PR, 7 SD, and 2 PD. No patients had CR. The ORR and DCR were 40.0% (95% CI 16.3%–67.7%) and 86.7% (95% CI 59.5%–98.3%), respectively. The DOR was not reached (range 0–544 days) in 5 patients (83.3%) with continued response at last follow-up. Furthermore, patients who received liver-directed radiotherapy had more PR (4 patients out of 8, 50%) than those who received non liver-directed radiotherapy (2 patients out of 7, 28.6%).

**Table 5:** Response evaluation.

Cohort	Value
All (n=15)	
CR	0
PR	6(40%)
SD	7(46.7%)
PD	2(13.3%)
ORR(95%CI)	40%(16.3%-67.7%)
DCR(95%CI)	86.7%(59.5%-98.3%)
DOR(median)	Not reached
Ongoing objective response at data cutoff (%)	5(83.3%)
Liver-directed radiotherapy (n=8)	
PR	4(50%)
SD	2(25%)
PD	2(25%)
Non liver-directed radiotherapy (n=7)	
PR	2(28.6%)
SD	5(71.4%)

CR, complete response; PR, partial response; SD, stable disease; PD, progressive disease; ORR, objective response rate; DCR, disease control rate; DOR, duration of response.

**Figure 1:**
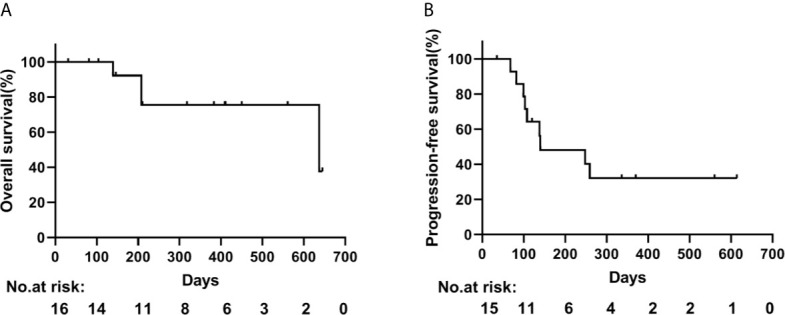
Kaplan-Meier curves of **(A)** overall and **(B)** progression-free survival.

By the cut-off date in January 2021, the median duration of follow-up was 383 days (range 31–645 days). A total of 4 deaths were observed among 16 patients (25.0%), and progression was found in 9 out of 15 cases (60.0%). Kaplan-Meier curves for OS and PFS were constructed (**Figure 1**). Patients had a median OS of 637 days (95% CI 27–1247 days), and the estimated rates of survival at 6 and 12 months were 92.3% and 75.5%, respectively. In addition, the median PFS was 140 days (95% CI 0–324 days). After a median follow-up of 411 days (range 81–645 days) among patients with liver-directed radiotherapy (n=8), 1 patient died. The median OS was 637 days, and the 12-month survival rate was 100%. However, there were 3 deaths in the remaining patients of non liver-directed radiotherapy (n=8), with a median OS of 208 days and 12-month survival rates of 50% (*P*=0.051). Furthermore, the median PFS for liver-directed radiotherapy (4 patients out of 8) was 259 days compared to 103 days (5 patients out of 7) for non liver-directed radiotherapy (*P*=0.236). The survival and time to PD are presented in **Figure S1**.

## Discussion

To the best of our knowledge, this is the first reported study that has combined PD-1**/**PD-L1 inhibitors, palliative radiotherapy, and anti-angiogenic therapy in HCC of BCLC stage C with a poor prognosis. Our preliminary findings have shown that triple therapy is safe in this patient population. Palliative radiotherapy may augment the anti-tumor activity of PD-1**/**PD-L1 blockade plus targeted angiogenesis agents, without unexpected toxicity.

A growing body of evidence suggested that concurrent radiotherapy plus immune checkpoint blockade was safe and feasible in cancer patients (22–24). However, it was unclear whether radiotherapy can be safely administered to patients also receiving the PD-1/PD-L1 inhibitors and targeted therapy combination. It should be noted that the safety profile of triple therapy in this population was similar to those reported in previous studies of two-drug combination therapy (8–10). In our study, the most common TRAEs were rash, diarrhea, aspartate aminotransferase increase, alanine transaminase increase, decreased appetite, and fatigue. Toxicity reached grade 3 or 4 in only 25% of patients, without treatment-associated mortality. No new safety signals or additive toxic effects were identified. In our study, laboratory abnormalities of hepatic events tended to occur more frequently in patients with liver-directed radiotherapy, but most were Grade 1–2 and manageable. Additionally, gastrointestinal hemorrhage, a severe complication in patients with HCC, was not observed in the liver-directed radiotherapy group and was found in only 1 patient with non liver-directed radiotherapy. Overall, the combination of triple therapy was tolerable with a manageable safety profile. Similarly, results from a Phase 1 study showed that the combination of hypofractionated stereotactic irradiation with pembrolizumab and bevacizumab in patients with recurrent high-grade glioma is generally safe and well tolerated (20).

The ORR of 40% in our cohort was promising. In CheckMate-040 and Keynote-224 studies, anti-PD-1 therapy resulted in an ORR of 15–20% among advanced HCC patients (2, 3). The combination of atezolizumab plus bevacizumab had an ORR of 27.3% according to independent assessment with RECIST 1.1 in IMbrave150 trial (8). In a Phase 1b study of lenvatinib plus pembrolizumab for unresectable HCC, the ORR was 36% according to RECIST 1.1 (9). Xu et al. reported that camrelizumab combined with apatinib had an ORR of 22.5–34.3% in the RESCUE trial (10). Prior studies included not only HCC of BCLC stage C but also of stage A and stage B. However, HCC of BCLC stage C with a larger tumor burden (portal vein invasion and**/**or extrahepatic spread) and poorer baseline characteristic in our study was enrolled. Additionally, the anti-tumor activity was long-lasting among the responders, which led to favorable survival outcomes with a median OS of 637 days. Lee et al. reviewed (25) that radiotherapy enhanced immune infiltrates into the tumor microenvironment (TME) but induced upregulation of immune checkpoint molecules (PD-1, PD-L1) and VEGF. Moreover, anti-VEGF therapy promoted normalization of vessel formation, which improved the efficacy of radiotherapy and further boosted infiltration of cytotoxic cells into TME. Therefore, a synergistic combination with triple therapy may further enhance anti-tumor immune responses. However, our study was small size and retrospective, which precluded an ability to determine if radiotherapy improved the efficacy of immune checkpoint blockade and anti-angiogenesis agents.

Currently, radiotherapy is turning into a crucial component of multidisciplinary treatment for HCC. There have been reports that radiotherapy-induced immune effect lasts for a short duration (26, 27). Therefore, radiotherapy was administered concurrently, starting within 14 days of the first dose of PD-1**/**PD-L1 inhibitors in this study. As expected for patients with HCC, the most irradiated site was the liver. In our study, the combined strategy seemed to be highly effective in liver-directed radiotherapy, with an ORR of 50% and a 12-month survival rate of 100%. Yu et al. reported that liver-directed radiotherapy eliminates immunosuppressive hepatic macrophages, increases hepatic T cell survival, and reduces hepatic siphoning of T cells. The combination of liver-directed radiotherapy and immunotherapy could promote systemic antitumor immunity (28). Furthermore, palliative liver-directed radiotherapy resulted in a clinically meaningful improvement in symptoms for advanced HCC patients with pain or abdominal discomfort and has tolerable toxicity (29, 30).

This study is limited by its small sample size and retrospective nature. In addition, a potential selection bias may exist due to the small number of cases using triple therapy in clinical practice. Moreover, the substantial heterogeneity of the patient population and treatment regimen may affect the interpretation of our findings. Thus, it is difficult to draw a valid conclusion about the efficacy of triple therapy. Prospective studies are needed to determine whether combining PD-1**/**PD-L1 inhibitors, anti-angiogenic agents, and radiotherapy potentiates efficacy.

## Conclusions

In conclusion, radiotherapy did not markedly increase the occurrence of side effects induced by PD-1**/**PD-L1 inhibitors and anti-angiogenic agents. The combination of triple therapy was safe and feasible in HCC of BCLC stage C. Our study provides a rationale to conduct a prospective trial to test the addition of radiotherapy to checkpoint inhibitor plus targeted angiogenesis therapy for advanced HCC. Such a study was recently developed (No. CXPJJH11900001-2019210) (31). Future studies may focus on more effective plans of each approach and an optimal combination to improve the efficacy of refractory HCC.

## Data Availability Statement

The raw data supporting the conclusions of this article will be made available by the authors, without undue reservation.

## Ethics Statement

The studies involving human participants were reviewed and approved by Medical Ethics Committee of Nanfang Hospital Southern Medical University and Medical Ethics Committee of Ganzhou People’s Hospital. Written informed consent for participation was not required for this study in accordance with the national legislation and the institutional requirements.

## Author Contributions

DW and LZ: study design and manuscript writing. WP: statistical analysis. HS and YX: data elaboration and manuscript revision. XZ and YW: data collection. All authors contributed to the article and approved the submitted version.

## Funding

This work was supported by the Science and Technology Project Foundation of Ganzhou City (GZ2019ZSF190).

## Conflict of Interest

The authors declare that the research was conducted in the absence of any commercial or financial relationships that could be construed as a potential conflict of interest.

## Supplementary Material

Supplementary Figure S1 Swimmers plot detailing survival for patients with liver-directed radiotherapy (n=8) and non–liver-directed radiotherapy (n=8). Bar length corresponds to the survival time.

## Abbreviations

AFP, alpha-fetoprotein; BCLC, Barcelona clinic liver cancer; CR, complete response; DCR, disease control rate; DOR, duration of response; ECOG, Eastern Cooperative Oncology Group; HBV, hepatitis B virus; HCC, hepatocellular carcinoma; ICI, immune checkpoint inhibitor; NSCLC, non–small cell lung cancer; ORR, objective response rate; OS, overall survival; PD, progressive disease; PD-1**/**PD-L1, programmed death receptor-1**/**programmed death ligand-1; PFS, progression-free survival; PR, partial response; SBRT, stereotactic body radiotherapy; SD, stable disease; TRAE, treatment-related adverse events.
