# Supplementary figures and images for: Safety of PD-1/PD-L1 Inhibitors Combined With Palliative Radiotherapy and Anti-Angiogenic Therapy in Advanced Hepatocellular Carcinoma
Source: Front Oncol. 2021 May 19;11:686621. doi: 10.3389/fonc.2021.686621 (PMC8170410; doi:10.3389/fonc.2021.686621)

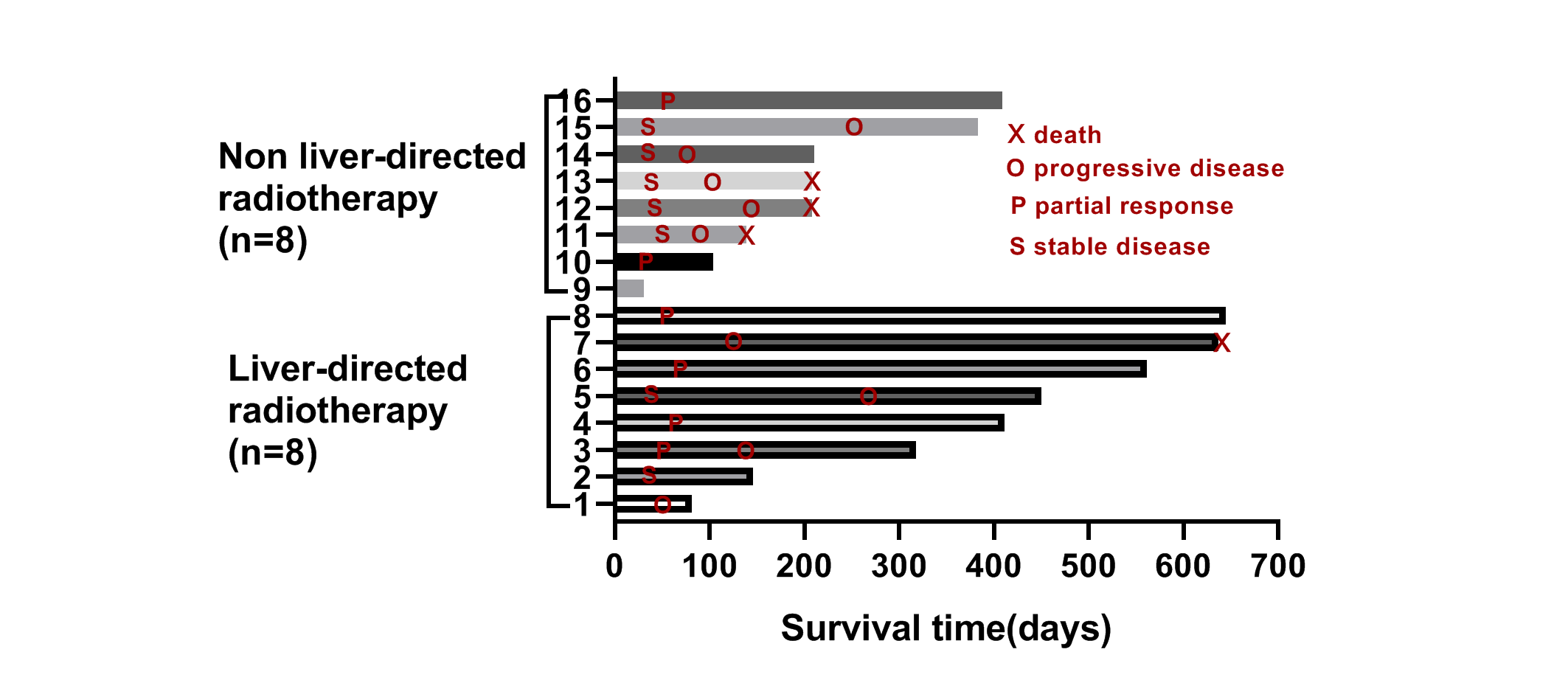

Supplement: Supplementary Figure S1 — Swimmers plot detailing survival for patients with liver-directed radiotherapy (n=8) and non–liver-directed radiotherapy (n=8). Bar length corresponds to the survival time. [file Image_1.tif]
