# Supplementary material for: Safety of PD-1/PD-L1 Inhibitors Combined With Palliative Radiotherapy and Anti-Angiogenic Therapy in Advanced Hepatocellular Carcinoma
Source: Front Oncol. 2021 May 19;11:686621. doi: 10.3389/fonc.2021.686621 (PMC8170410; doi:10.3389/fonc.2021.686621)
Supplement: Supplementary file 2 [file Table_1.doc]

Table S1. Treatment Related Adverse Events in liver-directed radiotherapy group and non liver-directed radiotherapy group.

| Adverse Events | Liver-directed radiotherapy (n=8) | | Non Liver-directed radiotherapy (n=8) | |
| --- | --- | --- | --- | --- |
|  | Any Grade n(%) | Grade3/4 n(%) | Any Grade n(%) | Grade3/4 n(%) |
| Rash | 3(37.5%) | 1(12.5%) | 1(12.5%) | 0 |
| Diarrhea | 2(25.0%) | 0 | 2(25.0%) | 0 |
| Aspartate aminotransferase increase | 2(25.0%) | 1(12.5%) | 1(12.5%) | 0 |
| Alanine transaminase increase | 2(25.0%) | 0 | 1(12.5%) | 1(12.5%) |
| Decreased appetite | 2(25.0%) | 0 | 1(12.5%) | 0 |
| Fatigue | 1(12.5%) | 0 | 2(25.0%) | 0 |
| Pruritus | 1(12.5%) | 0 | 1(12.5%) | 0 |
| Dental ulcer | 0 | 0 | 2(25.0%) | 0 |
| Nausea | 1(12.5%) | 0 | 1(12.5%) | 0 |
| Hypertension | 1(12.5%) | 0 | 0 | 0 |
| Infusion-related reaction | 1(12.5%) | 0 | 0 | 0 |
| Gastrointestinal hemorrhage | 0 | 0 | 1(12.5%) | 1(12.5%) |
| Blood bilirubin increase | 1(12.5%) | 0 | 0 | 0 |
| Weight decrease | 0 | 0 | 1(12.5%) | 0 |
